# Suicide by Proxy: A Psychoanalytic Hypothesis Illustrated by Three Forensic Case Reports

**DOI:** 10.1155/crps/6643923

**Published:** 2026-09-26

**Authors:** Yasir Karkosh, Badar Al Habsi, Salim Al-Huseini

**Affiliations:** ^1^ Department of Forensic Psychiatry, Al Masarra Hospital, Ministry of Health, Muscat, Oman, moh.gov.om; ^2^ Department of Rehabilitation Psychiatry, Al Masarra Hospital, Ministry of Health, Muscat, Oman, moh.gov.om

**Keywords:** case series, forensic psychiatry, Freudian perspective, homicide, suicide by proxy

## Abstract

This paper proposes the hypothesis of Suicide by Proxy, a psychodynamic formulation suggesting that certain acts of homicide may represent displaced manifestations of suicidal intent. Drawing on three retrospectively analysed forensic psychiatric cases involving individuals with psychotic disorders and documented suicidal ideation, this study illustrates how, from a psychoanalytic perspective, unresolved Oedipal dynamics, harsh punitive superego processes and internalised aggression may contribute to homicidal behaviour. The cases are interpreted as compatible with the hypothesis that, when direct self‐harm is psychologically inhibited, suicidal impulses may be redirected towards a symbolic other, such that homicide may function as a proxy for self‐destruction. Although based on a small, purposively selected case series and therefore hypothesis‐generating rather than confirmatory, this formulation offers a novel perspective on the relationship between suicidality and homicidal behaviour. Rather than proposing a new diagnostic entity or established causal mechanism, the study presents a psychodynamic hypothesis intended to stimulate further clinical and empirical investigation of the relationship between self‐directed and other‐directed aggression.

## 1. Introduction

Understanding the relationship between suicide and homicide has long been a subject of interest in psychiatry, psychology and the social sciences. Durkheim [1] observed that suicide and homicide sometimes coexist, sometimes vary inversely, and at other times respond similarly to social conditions, highlighting the complex relationship between these two forms of violence. Later, Henry and Short [2] conceptualised suicide and homicide as differing expressions of aggression, with suicide representing aggression directed inwards and homicide representing aggression directed outwards.

More recent research has demonstrated important clinical links between suicidality and interpersonal violence. Prior research has also identified an association between violent behaviour and completed suicide, further supporting an overlap between self‐ and other‐directed violence [3]. In a study of 210 forensic psychiatric evaluations of individuals who committed homicide, Richard‐Devantoy et al. [4] found that depression, delusional symptoms and suicidal ideation were more frequent among offenders with major mental disorders than among those without major mental disorders. Likewise, studies of homicide‐suicide have identified depression, psychosis and substance misuse among the psychiatric conditions commonly associated with these acts [5–7]. Furthermore, longitudinal evidence suggests that violence and suicidality exhibit a bidirectional relationship across development, indicating that each may increase the risk of the other [8]. Despite these observations, relatively little attention has been given to the possibility that, in some individuals, suicidal processes may contribute to homicidal behaviour even when suicide itself does not occur.

Menninger [9] conceptualised suicide as comprising three distinguishable psychological components: the wish to kill, the wish to be killed and the wish to die, emphasising that these components may coexist with different degrees of intensity within the same individual. He further observed that some individuals may wish to die yet remain unable to carry out a direct suicidal act. This observation suggests the possibility of an underlying conflict between destructive wishes and psychological forces opposing direct self‐destruction. It is particularly relevant to the cases examined in the present report, in which suicidality preceded the homicidal or attempted homicidal behaviour, yet direct suicide was not completed. The present hypothesis considers whether, in some such cases, this unresolved conflict might contribute to the redirection of self‐destructive aggression towards another person.

A related phenomenon is extended suicide, a form of homicide‐suicide in which an individual’s suicidal process encompasses the death of another person, typically a close family member. The perpetrator’s own death remains central to the act, while the victim may be incorporated into the suicidal intention through altruistic, pseudo‐altruistic, dependent or psychopathologically distorted motives. Extended suicide has been particularly associated with severe depressive illness, although psychotic and other psychiatric conditions may also occur [10, 11]. The present hypothesis shares with extended suicide the possibility that self‐ and other‐directed destructive impulses may arise within the same psychopathological process but differs in one fundamental respect: in Suicide by Proxy, direct suicide is not completed and is hypothesised to remain psychologically inhibited. Rather than taking another person along in an intended shared death, self‐destructive aggression is proposed to become redirected towards an external victim, who may acquire symbolic significance within the patient’s internal conflict. Thus, extended suicide provides an important neighbouring construct while remaining conceptually distinct from the phenomenon proposed here.

One theoretical perspective through which this possibility may be explored is the psychoanalytic theory. Freud [12] proposed that aggressive impulses may be directed either outward towards others or inward towards the self. In Civilization and Its Discontents, he argued that aggression inhibited by the demands of civilisation may become internalised through the action of the superego, contributing to guilt and self‐punishment [12]. In Mourning and Melancholia, Freud [13] further suggested that hostility towards a lost or disappointing object may be redirected against the self, producing severe self‐reproach and suicidal tendencies. From a psychoanalytic perspective, these formulations suggest that aggression is dynamic rather than fixed and may be redirected between self and others under different psychological conditions.

Object relations theory provides an additional framework for understanding the internalisation of the interpersonal experience. In Kernberg’s formulation, internalised object relations involve self‐representations and representations of significant others (object‐representations) linked by the affective experience. Psychological integration involves the capacity to incorporate contradictory positive and negative experiences into relatively coherent representations of both self and others. In less integrated functioning, these representations may remain divided, such that the self may be experienced alternately as worthy and successful or as entirely failed and worthless, while the same significant other may be experienced as either caring and admirable or rejecting and critical. Kernberg [14] further proposed that severe psychopathology may involve impaired integration and, at the psychotic level, impaired differentiation between self‐ and object‐representations, together with loss of reality testing.

His formulation therefore provides a theoretical basis for considering how self‐ and object‐representations may become projected onto external persons. Whether such processes contribute to victim selection or homicidal behaviour, however, remains a hypothesis in the present study.

Impulsivity represents an important alternative or complementary explanation for both self‐directed and interpersonal violence. It has frequently been proposed as a mechanism underlying violent behaviour in psychosis; however, the available evidence does not support impulsivity as a sufficient explanation for such behaviour. Bjørkly’s [15] systematic review of the relationship between impulsivity and violence in psychotic disorders found limited and inconclusive evidence and cautioned against assuming a simple causal relationship. Similarly, a systematic review and meta‐analysis by Moore et al. [16] found only a weak overall association between impulsivity and suicidality, with substantial heterogeneity across studies, suggesting that impulsivity alone cannot adequately account for suicidal behaviour.

These findings suggest that, although impulsivity may contribute to the execution or timing of violent acts, it may not adequately explain why a suicidal individual with psychosis directs lethal aggression towards another person rather than towards the self. The present hypothesis, therefore, does not rule out a contributory role for impulsivity; rather, impulsivity may function as a precipitating or facilitating factor in the behavioural expression of aggression. The proposed psychodynamic formulation addresses a different question: whether previously established self‐directed aggression may, under particular psychological conditions, become externalised towards another person who comes to symbolise the patient’s internal conflict. In this sense, the present formulation is intended to complement, rather than replace, existing models of psychotic violence by considering the potential psychological direction of aggression in addition to its behavioural expression.

Contemporary models similarly emphasise that both violence in psychosis and suicidal behaviour are multifactorial phenomena that cannot be attributed to a single psychological mechanism. Research on violence in psychosis identifies interacting clinical, historical and contextual factors, including previous violence or criminal behaviour, substance misuse, active psychopathology, treatment‐related factors and psychosocial adversity [17, 18]. Modern theories of suicidal behaviour likewise distinguish the development of suicidal ideation from the transition to suicidal action and emphasise interactions among psychological pain, hopelessness, entrapment, connectedness and factors influencing the capacity to act on suicidal thoughts [19, 20]. Accordingly, the psychodynamic formulation proposed here is not intended to replace these established perspectives. Rather, it considers whether unconscious processes involving internalised object relations, unresolved Oedipal dynamics, projection and displacement might provide an additional explanation for the direction of aggression in a small subgroup of suicidal individuals with severe mental illness.

Building on Freud’s [12, 13] account of aggression being redirected from an external object towards the self, the present paper asks whether, under particular circumstances, the reverse process might also occur: could an individual with severe suicidal impulses who is unable to complete suicide redirect aspects of those self‐destructive impulses towards another person? Rather than proposing this as an established mechanism, we present it as a hypothesis that may offer one possible psychodynamic explanation for a small subset of homicidal acts.

To explore this possibility, we present three forensic psychiatric cases involving individuals with documented suicidality, severe psychotic or affective illness and homicide or attempted homicide. We propose the term ‘Suicide by Proxy’ to describe the hypothesis that, in certain individuals, homicidal behaviour may function as a symbolic displacement and external enactment of otherwise inhibited self‐destructive impulses. This formulation is hypothesis‐generating and is not intended to establish a new diagnostic construct, a validated causal mechanism, or a universally applicable explanation for homicidal behaviour. Rather, the cases are presented to illustrate the proposed formulation and to generate questions that may subsequently be examined through systematic empirical investigation.

## 2. Method

Three forensic psychiatric cases were retrospectively analysed through a psychodynamic lens. The cases were purposively selected from available forensic psychiatric evaluations because they demonstrated clinical features relevant to the proposed hypothesis, including documented psychosis or severe mood disturbance, a history of suicidal ideation or suicidal behaviour preceding the offence and subsequent homicide or attempted homicide. These cases were selected for their illustrative value in exploring the possible relationship between self‐directed and other‐directed aggression rather than as consecutive cases or as a representative forensic psychiatric sample. Accordingly, the findings should be interpreted as hypothesis‐generating rather than confirmatory. The possibility of selection bias is acknowledged, and these cases cannot be used to estimate the prevalence, frequency or specificity of the proposed phenomenon.

Clinical information was obtained from forensic psychiatric interviews, collateral information, medical records, legal documentation and available psychological assessments, including the Minnesota Multiphasic Personality Inventory (MMPI) and structured inventory of malingered symptomatology (SIMS), where applicable. The case descriptions were derived from clinical material obtained during forensic psychiatric evaluations. All identifying information was removed or modified to preserve confidentiality, while maintaining the clinical characteristics relevant to the formulation and interpretation of each case.

### 2.1. Case Presentations

#### 2.1.1. Case 1: Murder of Foreign Man

Mr. K was a 26‐year‐old man with no previous psychiatric history who, prior to the onset of his illness, was described by his family as a caring, socially well‐integrated and popular individual within his community. He had strong academic ambitions and was actively engaged in several hobbies.

In 2013, during his second year of college, Mr. K experienced a gradual decline in academic performance and social functioning. He became increasingly withdrawn, spent prolonged periods daydreaming and demonstrated inappropriate affect, including episodes of laughing without an apparent stimulus. His functional decline was accompanied by increasing self‐deprecatory thoughts and a growing sense of alienation. During this period, a history of cannabis and methamphetamine use was identified, and his mental state further deteriorated following the imprisonment related to substance use. A significant family tragedy occurred around the same period, followed by social rejection and ostracisation from some extended family members, which appeared to contribute to further psychosocial deterioration. Over the following years, Mr. K developed persistent psychotic symptoms, including paranoid delusions that he was being monitored by military forces who were attempting to prevent his academic and professional success. He also developed bizarre beliefs regarding his identity, including the conviction that he was of foreign origin rather than his actual nationality. He reported olfactory hallucinations, particularly smelling of supernatural beings (jinn), and believed that he was under their influence. His behaviour became increasingly unusual, including burning official documents, purchasing weapons and subsequently discarding them and withdrawing socially for prolonged periods.

Three days before the incident, Mr. K purchased a new firearm, which resulted in another confrontation with his father, who was a police officer. On the day of the homicide, after another argument with his father, he left his home and entered a nearby mosque and shot a man who was praying. Mr. K has no previous relationship with the victim nor any identifiable personal motive to kill him.

Following the incident, he placed the firearm beside the victim and returned home, where he slept. He denied being intoxicated or under the influence of substances at the time of the offence. During the initial psychiatric assessments, Mr. K denied suicidal thoughts at the time of the incident. However, after treatment with antipsychotic medication and a reduction of his guardedness, he disclosed a longstanding history of suicidal thoughts and several previous suicide attempts. He reported that the firearm purchased before the incident had originally been intended for suicide, although he had been unable to act on this intention. During this interview, he became deeply distressed and cried while describing his previous suicidal experiences.

Mr. K was diagnosed with schizophrenia, characterised by prominent persecutory symptoms. His history indicated longstanding untreated psychotic symptoms, preceded by substance misuse and progressive behavioural changes over several years before the offence. Unfortunately, he had not received psychiatric assessment or treatment before the homicide. During the admission, he was treated with risperidone 6 mg and citalopram 20 mg. Following treatment, his psychotic symptoms and mood improved significantly, and his suicidal thoughts were reduced. He continued psychiatric follow‐up and remained adherent to treatment.

#### 2.1.2. Case 2: Attempted Murder of the Father

Mrs. B was a 39‐year‐old married woman and the sixth of nine siblings (four brothers and five sisters). She was raised in a family in which her father, previously a simple farmer, was described as having a peaceful and submissive personality, while her mother was perceived as the dominant figure within the household. Mrs. B described her mother as strict and emotionally harsh, with a perceived preference towards sons and differential treatment of daughters. She reported longstanding feelings of unfairness and inequality within the family, describing a division between her brothers, who were perceived as supported by their mother, and the daughters, who felt marginalised. Throughout her childhood and adolescence, Mrs. B developed low self‐esteem, which was further affected by poor academic achievement and repeated criticism from teachers regarding her failure to meet expectations. After marriage, she described her disappointment with her husband, who worked as a school cleaner and whom she perceived as having a passive and weak personality. During psychiatric interviews, she expressed resentment towards him because of his perceived inability to protect her or challenge her brothers. She reported that she had always attempted to defend herself and her sisters’ rights and felt strongly motivated to correct what she perceived as a longstanding injustice within the family.

Her mental state deteriorated following a significant family conflict. During an argument, her brother assaulted her by slapping her when she attempted to report the incident to the police. However, her husband and father prevented her from pursuing legal action. Following this event, she developed severe depressive symptoms, including insomnia, reduced appetite, low mood and suicidal ideation. She also developed psychotic symptoms, including paranoid delusions that her family members were conspiring against her and that her brother intended to harm or kill her. During this period, she began sending messages to family members indicating that she might end her life, while simultaneously sending highly aggressive and insulting messages containing profanity and abusive language towards her older brothers. These behaviours were considered markedly inconsistent with her previously described shy and submissive personality.

Mrs. B was diagnosed with a severe major depressive episode with psychotic features. During this period, she developed a plan to end her life in the presence of her father. On the day of the incident, she took her children and went to spend the night at her father’s house. While the family members were asleep, she took a knife that she had brought from her own home and entered her father’s room, where he was sleeping on a couch. She removed the knife and, instead of using it on herself as she had intended, stabbed her father in the head. She immediately began crying and shouting, which alerted the family members, who intervened. At the time of intervention, she was still holding the knife and attempting to harm herself. Mrs. B had no previous psychiatric history. During her admission, she was treated with olanzapine 20 mg and fluoxetine 20 mg, with marked improvement in her depressive symptoms, psychotic symptoms and overall mental state.

#### 2.1.3. Case 3: Attempted Murder of Mother

Miss Z was a 36‐year‐old woman who lived with her parents and two sisters. Prior to the onset of her illness, she was highly motivated to pursue higher education, achieve personal independence, establish herself as a productive member of society and support her sisters in escaping the challenging family environment. Her father had a longstanding history of alcohol misuse and was described as abusive, irresponsible and frequently intoxicated. The family environment was characterised by recurrent parental conflict and significant emotional distress.

Miss Z reported feelings of shame towards her father and longstanding resentment towards her mother for remaining in the marriage. She believed that her mother had failed to protect her and her sisters from their father’s abusive behaviour. Despite these difficulties, the relationship between Miss Z and her mother was characterised by significant emotional complexity. They shared similar educational aspirations; her mother worked as a nursery teacher and continued her own education, while Miss Z also valued education, taught younger students in her spare time and attempted to protect her younger sister from their father’s abusive behaviour.

In 2010, Miss Z was assessed for involuntary jerky movements and was diagnosed with conversion disorder. After completing secondary school, she enrolled in a private college and remained strongly committed to her education. In 2016, she travelled abroad to continue her studies; however, during this period, she experienced increasing academic difficulties accompanied by psychological deterioration. She developed auditory hallucinations of unknown individuals inviting or commanding her to leave this world, which she interpreted as messages encouraging her to die. She also developed persecutory symptoms, including ideas or delusions of reference, beliefs that she was being monitored and thought broadcasting. During this period, she made several suicide attempts, including an attempt by hanging.

While abroad, she was diagnosed with schizophrenia and epilepsy and was treated with olanzapine and carbamazepine, resulting in clinical improvement. Although epilepsy was diagnosed, no definite epileptic seizures were documented in the subsequent clinical records. Following her inability to complete her education, a significant conflict developed between Miss Z and her mother. Her mother expressed disappointment regarding her academic failure and the financial resources invested in her education. Miss Z, however, attributed her difficulties partly to her mother’s earlier failure to protect her and her sisters from the adverse family environment.

After returning home, Miss Z discontinued her medication in 2021. Over the following years, her mental state progressively deteriorated, with increasing social withdrawal, recurrent auditory hallucinations, ideas or delusions of reference, thought broadcasting, beliefs that she was being monitored by unknown individuals, and increasing suspiciousness towards her mother. Her longstanding resentment towards her mother persisted, particularly regarding her perception that her mother had failed to protect the family and had criticised her educational failure.

During this period of relapse, Miss Z continued to experience suicidal thoughts and auditory hallucinations with commands to end her life. She concealed these experiences from her family and reported privately contemplating suicide. Approximately 3 days before the incident, she developed thoughts of killing her mother and placed a knife beneath her pillow.

On the day of the incident, while experiencing auditory hallucinations and significant emotional distress, Miss Z went to the bathroom. While looking at herself in the mirror, she became increasingly agitated, broke the mirror, and took a fragment of glass. She then approached her mother, who was sitting in the corridor, with the intention of attacking her. Her sister intervened and prevented the assault from being completed. During subsequent psychiatric assessment, Miss Z denied having a clear conscious intention to kill her mother immediately before the incident. She described longstanding feelings of resentment towards her mother, stating that she felt unloved, verbally mistreated and unsupported. She also reported that, at the time of the incident, she feared that her mother might harm her. Her suicidal thoughts and death‐related auditory hallucinations persisted during the initial assessment. In her statement to the police, Miss Z reported that she attempted to kill her mother because she believed her mother did not love her and had verbally and physically abused her. During later psychiatric interviews, however, she expressed affection towards her mother despite their conflicts and denied any ongoing wish to harm her. At that time, suicidal thoughts and auditory hallucinations commanding her to die remained prominent.

Miss Z was treated with carbamazepine and olanzapine, which were later switched to aripiprazole because of hyperprolactinaemia. Following treatment, she showed marked improvement in psychotic symptoms, including auditory hallucinations, as well as improvement in mood and insight. She no longer expressed homicidal intent towards her mother and demonstrated an improved understanding of the relationship between her symptoms and her behaviour.

## 3. Discussion

### 3.1. Psychodynamic Explanation of Mr. K

From a Freudian perspective, case 1 may represent a different psychodynamic pathway from cases 2 and 3. Rather than arising primarily from longstanding ambivalence towards an attachment figure, psychotic decompensation may have been accompanied by regression to repressed Oedipal conflicts, allowing previously repressed hostile wishes towards the paternal figure to re‐emerge. His father’s role as a police officer, together with persistent delusions of surveillance, monitoring and control by governmental authorities, may be understood as reflecting the symbolic persistence of paternal authority within his psychotic experience. These persecutory experiences may represent the projection of previously repressed hostility towards the paternal authority figure or, alternatively, the externalisation of a harsh punitive superego onto vague external authorities. At the same time, his repeated suicidal behaviour and chronic self‐destructive acts provide evidence of a prominent self‐destructive process. Within a Freudian formulation, this may be understood as aggression directed towards the self, potentially associated with guilt and self‐punishment arising from hostility that could not be consciously expressed towards the loved paternal object.

From an object‐relations perspective, psychotic decompensation may have weakened the differentiation between self‐ and object‐representations, allowing the internalised paternal representation to become increasingly intertwined with rejected aspects of the self. Within the present hypothesis, the unfamiliar victim may therefore have functioned simultaneously as a symbolic paternal object and as an extension of the patient’s rejected self, potentially allowing self‐directed aggression to become displaced outward. The choice of an unfamiliar victim within a mosque (house of God) may represent one possible symbolic enactment of this internal conflict. The mosque, as a place culturally associated with moral and paternal authority, may have provided a symbolic context for the paternal figure. The patient’s subsequent decision to leave the firearm beside the victim rather than conceal or remove it may likewise be interpreted in more than one way. If the victim functioned symbolically as the paternal object, the act may represent the expression of previously inhibited hostility towards that object. Alternatively, if the victim also functioned as a symbolic extension of the patient’s rejected self, leaving the weapon beside the body may represent an unconscious acknowledgement of guilt, remorse or symbolic surrender following the possible external enactment of self‐destructive aggression. Within the present hypothesis, these proposed symbolic meanings may not be mutually exclusive, as psychotic weakening of self–object differentiation may have allowed the victim to function simultaneously as both a paternal object and a representation of the rejected self. These interpretations remain speculative and cannot establish unconscious motivation or causation, nor can they exclude alternative explanations, including psychosis‐related violence without symbolic displacement.

Proposed psychodynamic pathway:

Regression to unresolved Oedipal conflict → aggression redirected towards the self → psychotic weakening of self–object differentiation → paternal representation becomes intertwined with rejected self → unfamiliar victim may simultaneously represent paternal object and rejected self → self‐directed aggression may be displaced onto the victim.

### 3.2. Psychodynamic Explanation of Mrs. B

From a Freudian perspective, Mrs. B’s enduring attachment to her father as the primary paternal love object within the positive Oedipal relationship, despite his repeated failure to protect her from her harsh and rejecting mother, may have generated unresolved ambivalence. Because hostility towards such a loved object could not be fully expressed within a Freudian formulation, this hostility may have been redirected towards the self, potentially contributing to her self‐destructive wishes and behaviour.

From an object‐relations perspective, her internal representation of her father may have remained split between an idealised and a disappointing object, resulting in persistent ambivalence. In case‐specific terms, these representations may be understood as involving a vulnerable and rejected self‐representation (‘the daughter who was not protected’) and a disappointing paternal representation (‘the father who failed to protect her’). Within the present hypothesis, psychotic decompensation may have weakened the differentiation between self‐ and object‐representations, such that the disappointing paternal representation may have become increasingly intertwined with rejected aspects of the self. The father may consequently have been experienced simultaneously as the disappointing paternal object and as a symbolic extension of the patient’s vulnerable and rejected self, potentially allowing self‐directed aggression to become displaced towards him. This interpretation remains speculative and cannot establish unconscious motivation or causation, nor can it exclude alternative explanations for the assault, including psychosis‐related violence, interpersonal hostility and impaired behavioural control.

Proposed psychodynamic pathway:

Split paternal representation → psychotic weakening of self–object differentiation → disappointing father may become intertwined with rejected self → self‐directed aggression may be displaced onto father.

### 3.3. Psychodynamic Explanation of Miss Z

From a Freudian perspective, Miss Z’s enduring emotional attachment to her mother as the primary maternal object of identification, despite her perceived failure to protect her from her abusive father and her subsequent criticism following academic failure, may have generated unresolved ambivalence. Because hostility towards such an important attachment figure could not be fully expressed within a Freudian formulation, this hostility may have been redirected towards the self, potentially contributing to her longstanding suicidality.

From an object‐relations perspective, her representation of her mother may have remained split between an admired and identified‐with caregiving figure who represented the person she aspired to become and a critical, disappointing object who had failed to protect her. Following the collapse of her academic aspirations, her own sense of failure may have consolidated a negative self‐representation centred on inadequacy and shame. According to Kernberg’s [14] object‐relations formulation, psychotic decompensation may involve a regressive weakening of differentiation between self‐ and object‐representations, together with impaired reality testing. Within the present hypothesis, this process may have allowed her negative self‐representation (‘I failed’) to become increasingly fused with her disappointing maternal representation (‘you failed me’), such that the mother may have become associated with rejected or failed aspects of the patient’s self. Through projection, these intolerable aspects of the self may have been externalised onto the maternal figure, potentially allowing aggression that had previously been directed towards the self to become displaced towards her. The attempted assault may therefore be understood as a possible symbolic external enactment of an underlying self‐destructive conflict. This interpretation remains speculative and cannot establish unconscious motivation or causation, nor can it exclude alternative explanations for the assault, including psychosis‐related violence, persecutory ideation, interpersonal hostility and impaired behavioural control.

Proposed psychodynamic pathway:

Split maternal representation → psychotic weakening of self–object differentiation → disappointing mother may become intertwined with rejected/failed self → self‐directed aggression may be displaced onto mother.

Miss Z represents a more ambiguous example of the proposed formulation. Her longstanding suicidality, previous suicide attempt, persistent death‐related hallucinations and continuing suicidal wishes establish a prominent self‐destructive process preceding and accompanying the period of homicidal ideation. However, she had also developed thoughts of killing her mother approximately 3 days before the incident and described resentment towards her mother’s criticism and perceived lack of affection. These features provide a plausible alternative explanation of the assault as psychosis‐related violence arising from interpersonal hostility and impaired behavioural control.

Of particular relevance, the homicidal and suicidal processes did not follow identical trajectories. In her statement to the police following the incident, Miss Z attributed her attempt to kill her mother to interpersonal grievances, reporting that her mother did not love her, verbally abused her and physically assaulted her. During subsequent psychiatric assessment, however, she acknowledged that she loved her mother, although she continued to describe her as having treated her poorly and denied any continuing intention to kill her. In contrast, suicidal wishes and auditory hallucinations commanding her to kill herself persisted. Thus, mother‐directed homicidal intent appeared relatively circumscribed, whereas the underlying self‐destructive process remained active. Although the subsequent resolution of homicidal intent does not exclude an interpersonal, psychotic or persecutory contribution to the assault, the divergence between the two trajectories is noteworthy. This temporal pattern cannot establish that the homicidal impulse represented displaced suicidality, but it is compatible with the possibility that a persistent self‐directed aggressive process became temporarily externalised towards a psychologically significant figure during psychotic decompensation. The presence of victim‐directed persecutory ideation does not necessarily exclude the proposed formulation. In Miss Z’s case, the belief that her mother might harm her did not appear to constitute an entirely novel or psychologically unrelated persecutory theme but showed thematic continuity with a longstanding maternal conflict involving perceived rejection, criticism, lack of affection and failure to protection. Within the present hypothesis, psychotic decompensation may therefore have amplified an established interpersonal and intrapsychic conflict to a persecutory intensity. In such cases, victim‐related persecutory content might represent an intensified psychotic expression of a pre‐existing conflict that had also become associated with self‐directed aggression, rather than a wholly independent pathway to victim selection. This interpretation remains speculative and does not exclude persecutory psychosis as an alternative or contributing explanation for the assault.

This case illustrates both the potential relevance and limitations of the proposed model. Although persistent suicidality and the psychological significance of the victim are compatible with the Suicide by Proxy formulation, pre‐existing homicidal ideation, maternal resentment, psychotic decompensation and impaired behavioural control provide plausible alternative explanations. The case therefore cannot establish displacement of suicidal aggression; rather, it raises the possibility that self‐ and other‐directed aggression became intertwined within the same psychopathological process.

A psychodynamic question raised by these cases is why suicide, despite being repeatedly contemplated and, in some instances, previously attempted, was ultimately not completed. One possible explanation is that, although all three patients experienced intense self‐destructive impulses, they appeared to retain a residual psychological investment in preserving the self. Each patient had previously maintained ambitious aspirations and idealised expectations regarding his or her future despite longstanding adversity. The first patient aspired to exceptional academic achievement and to becoming a respected and caring figure within his community before the onset of psychosis; the second longed for dignity, fairness and a life fundamentally different from her lived experience; and the third pursued higher education and personal independence despite severe familial dysfunction and chronic social disadvantage. These aspirations appeared to provide psychological meaning, coherence and hope despite adverse developmental experiences.

The repeated frustration and eventual collapse of these aspirations may be conceptualised as a profound narcissistic injury, disintegrating an idealised self‐representation. Such a collapse may intensify shame, guilt, self‐reproach and the activity of a punitive superego, thereby contributing to the development of severe self‐directed aggression and persistent suicidal wishes. Nevertheless, the persistence of ambitious aspirations before illness and the repeated failure to complete suicide despite ongoing suicidal ideation suggest that some psychological investment in the self may have remained intact. From this perspective, the central conflict may not simply have been between life and death, but between a punitive superego demanding self‐destruction and a residual libidinal investment in the self that resisted complete annihilation.

Within the present hypothesis, psychotic decompensation may alter the manner in which this conflict is expressed rather than eliminating it. As described by Kernberg [14], psychotic organisation is associated with a regressive weakening of differentiation between self‐ and object‐representations, together with impaired reality testing. Under these conditions, rejected aspects of the self may become increasingly intertwined with psychologically significant internalised object representations. Through projection, these unwanted aspects of the self may be externalised onto another person who symbolically represents either the rejected self, a significant internalised object, or both simultaneously. Displacement may then permit aggression that had previously been directed towards the self to become redirected towards this externalised representation.

**Figure 1:**
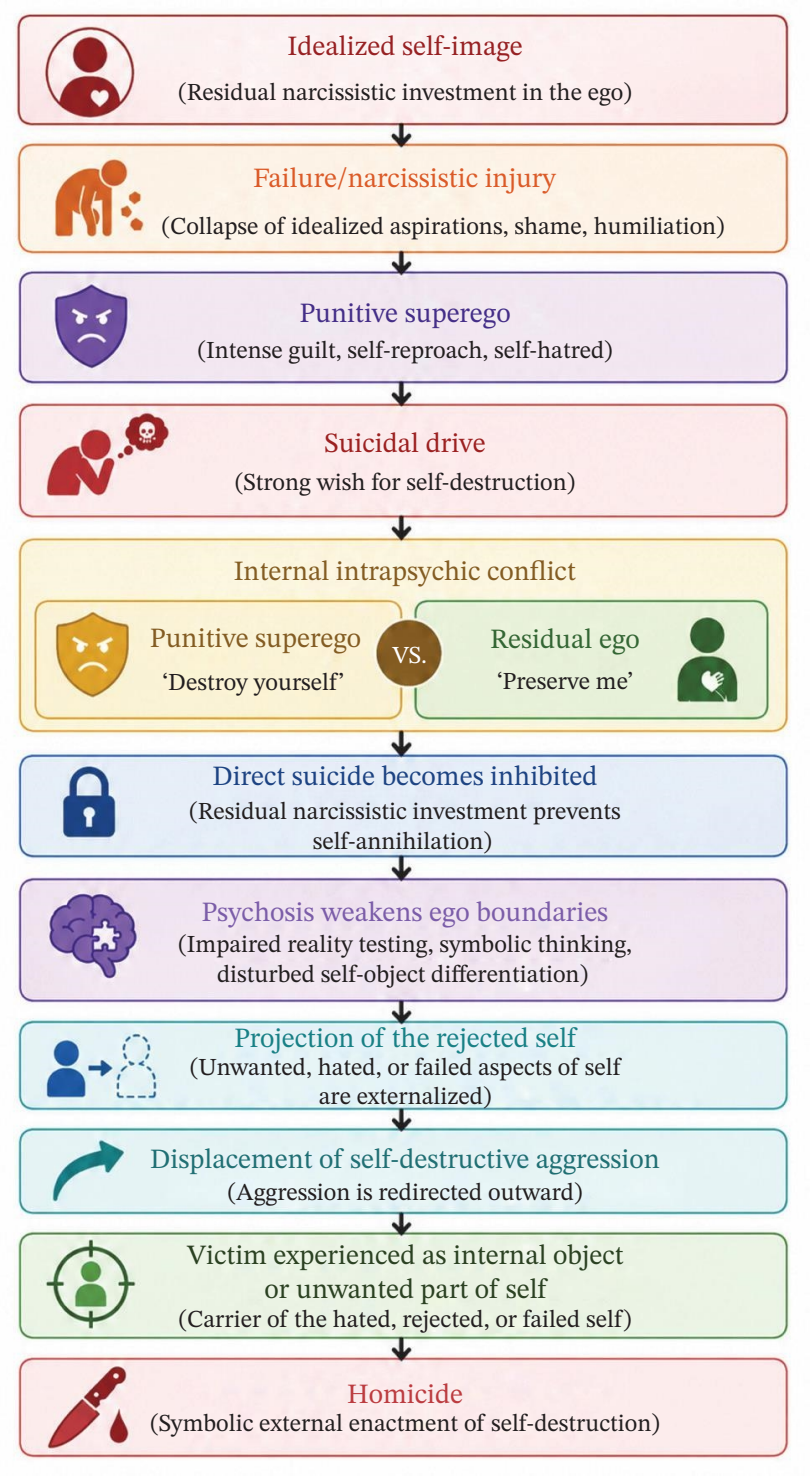
Conceptual psychodynamic model of the proposed suicide by proxy hypothesis. Following a narcissistic injury, a punitive superego generates intense self‐directed aggression and suicidal impulses. Because residual narcissistic investment in the ego preserves a libidinal attachment to the self, direct suicide becomes conflicted and inhibited. In the presence of psychosis, weakened ego boundaries facilitate projection of the rejected aspects of the self onto a symbolic other, allowing self‐destructive aggression to be displaced outward and enacted through homicide. This figure represents a hypothesis‐generating conceptual model rather than an empirically established causal pathway.

Accordingly, homicide, within this formulation, is conceptualised neither as simple outward aggression nor as merely an interrupted suicide. Rather, it may represent a symbolic external enactment of otherwise inhibited self‐destructive aggression directed towards a psychologically meaningful victim. The proposed mechanism therefore extends Freud’s concept of aggression redirected from the object towards the self by considering the possibility that, under conditions of psychotic decompensation and disturbed self‐object differentiation, self‐directed aggression may subsequently become redirected towards an external symbolic object; this proposed pathway is illustrated in Figure 1.

This interpretation remains speculative and should be regarded as one possible psychodynamic formulation rather than an established causal mechanism. Psychosis, impaired reality testing, persecutory delusions, impulsivity, neurobiological factors and psychosocial circumstances are all likely to have contributed to the violent behaviour observed in these cases.

The present hypothesis is intended to complement, rather than replace these established explanations. The proposed sequence is therefore offered as a conceptual psychodynamic model derived from the present cases rather than as a validated mechanism. Future research should examine whether these proposed processes distinguish psychotic offenders with antecedent suicidality from psychotic offenders who commit homicide without evidence of preceding self‐destructive intent.

### 3.4. Potential Implications for Forensic Psychiatry

The proposed Suicide by Proxy formulation raises the possibility that, in a subgroup of individuals with severe mental illness, self‐ and other‐directed aggression may be more closely related than traditionally conceptualised. If supported by future empirical research, this hypothesis could have potential relevance to understanding the relationship between suicidality, victim selection and violence in psychotic disorders. At present, however, the model should not be used to guide clinical or forensic decisions independently of established approaches to risk assessment and management.

Potential areas for future investigation include whether severe or persistent suicidality is associated with particular patterns of subsequent violence; whether the psychological relationship between the offender and victim contributes to the direction of aggression; and whether assessment of self‐directed aggression, guilt, shame and significant interpersonal conflicts adds predictive or explanatory value beyond established violence‐risk factors. Prospective and comparative studies would be required before such factors could be incorporated into routine violence‐risk assessment or clinical intervention.

If the Suicide by Proxy hypothesis is empirically supported, antecedent suicidality may represent not only a historical marker but a potentially important dynamic treatment target in a subgroup of psychotic offenders. In such cases, reduction of the underlying self‐destructive process might be associated with a reduction in the risk of its proposed externalisation as violence. This possibility requires prospective investigation and should not be interpreted as suggesting that resolution of suicidality alone is sufficient for discharge or that residual psychotic symptoms can be disregarded in the forensic risk assessment.

Similarly, although psychodynamic formulation may contribute to understanding the subjective meaning of an offence, the proposed Suicide by Proxy hypothesis should not currently be used to infer intent, motivation, criminal responsibility or diminished responsibility in individual forensic evaluations. Such determinations must remain based on the applicable legal standards and comprehensive assessment of the individual’s mental state, symptoms, diagnosis, cognitive capacities and relationship between the mental disorder and the alleged offence.

### 3.5. Provisional Features for Future Empirical Investigation

For future empirical investigation, a case may be considered potentially compatible with the proposed Suicide by Proxy hypothesis when the following features are present:

1. Antecedent suicidality: documented suicidal ideation, suicidal intent, suicide attempts or significant self‐destructive behaviour preceding the homicidal or attempted homicidal act.
2. Psychosis at the time of the offence: evidence of a psychotic disorder or clinically significant psychotic symptoms during the period in which the homicidal behaviour occurred.
3. Victim selection and relationship to the underlying suicidal conflict: the victim is not selected primarily because of a pre‐existing victim‐specific persecutory delusion in which the principal motive for violence is to eliminate an immediate or anticipated physical or psychological threat. If a pre‐existing victim‐related delusion is present, its content should show demonstrable continuity with the central psychological conflict underlying the offender’s antecedent suicidality, such as perceived failure, abandonment, rejection, disappointment, shame or failed protection. This continuity should, where possible, be supported by clinical history or collateral information predating the offence rather than be inferred retrospectively from the homicidal act alone.
4. The homicidal act should not be primarily explained by a conventional instrumental motive, such as financial gain, concealment of another crime or calculated revenge.

These features are proposed solely for future empirical investigation of cases that may warrant consideration within the Suicide by Proxy framework. They are not diagnostic criteria and should not be used to establish the psychological motivation for an offence, determine causation, assess criminal responsibility or guide individual clinical or legal decisions. Their reliability, specificity, discriminant validity and clinical significance remain unknown and require testing in independent samples, particularly through the comparison of psychotic individuals who commit serious violence with and without antecedent suicidality.

## 4. Conclusion

Suicide by Proxy is proposed as a hypothesis‐generating psychoanalytic framework for exploring the possible relationship between self‐ and other‐directed aggression in a subgroup of patients with severe mental illness. The present cases raise the possibility that, under conditions of psychotic decompensation, self‐destructive aggression may become displaced towards a psychologically significant victim and expressed as a symbolic external enactment of self‐directed aggression. This interpretation remains speculative and requires empirical investigation. Importantly, such a psychodynamic formulation should complement rather than replace comprehensive psychiatric assessment and treatment. Clinical understanding and management must remain grounded in the patient’s presenting symptoms, psychiatric diagnosis, longitudinal course of illness, suicide and violence risk and response to and adherence to treatment. Appropriate pharmacological treatment of the underlying disorder, together with indicated psychological, psychotherapeutic and psychosocial interventions, remains central to management and risk reduction. Further research is required to determine whether the proposed Suicide by Proxy formulation has explanatory or clinical value beyond established psychiatric and forensic models.

## Funding

No funding was received for this study.

## Ethics Statement

Ethical approval was waived by the Research Subcommittee of Al Masarra Hospital because this manuscript involved a retrospective review of anonymised clinical information obtained during routine forensic psychiatric evaluations and did not involve any additional intervention or risk to the patients.

## Consent

Written informed consent for publication was obtained from all three patients included in this case series. The manuscript contains no identifiable personal information, and all reasonable measures were taken to protect patient privacy and confidentiality. Identifying details were removed or modified where necessary without altering the clinical significance of the cases.

## Conflicts of Interest

The authors declare no conflicts of interest.

## Data Availability

The case report data used to support the findings of this study are included within the paper.
